# Differences in anti-inflammatory effect of immature and mature of *Rubus coreanus* fruits on LPS-induced RAW 264.7 macrophages via NF-κB signal pathways

**DOI:** 10.1186/s12906-019-2496-6

**Published:** 2019-04-25

**Authors:** Kyung Hye Seo, Ji Yeon Lee, Jeong-Yong Park, Gwi Yeong Jang, Hyung Don Kim, Young- Seob Lee, Dong Hwi Kim

**Affiliations:** 1 Department of Herbal Crop Research, National Institute of Horticultural & Herbal Science, Eumsung, 27709 Republic of Korea; 2 Department of Food Science and Biotechnology, Chungbuk National University, Cheongju, 28644 Republic of Korea

**Keywords:** Anti-inflammation, Free radical scavenging, Inflammatory cytokines, NF-κB signaling, *Rubus coreanus* fruit

## Abstract

**Background:**

*Rubus coreanus* fruit (RF) has been used as a traditional medicine formulation to treat various diseases including diarrhea, asthma, and cancer in East Asia (Korea, China, and Japan). RF, which is native to Korea, has a larger fruit size than that of exotic species. In this study, we aimed to compare the anti-inflammatory activities of immature and mature RF extracted with different solvents.

**Methods:**

Mature and immature RF (MRF and IRF) were extracted with 30% ethanol, 70% ethanol and water at room temperature. The antioxidant activity was determined using 2,2-diphenyl-1-picrylhydrazyl (DPPH) and 2,2′-azino-bis(3-ethylbenzothiazoline-6-sulphonic acid (ABTS) assays. Anti-inflammatory activity was determined by measuring nitric oxide (NO) production, expression of inflammatory proteins (inducible NO synthase [iNOS], cyclooxygenase [COX]-2, nuclear factor [NF]-κB, and inhibitor of NF-κB [IκB]), and inflammatory cytokines using polymerase chain reaction in lipopolysaccharide (LPS)-stimulated RAW264.7 macrophages.

**Results:**

The IRF 30% ethanol extract showed higher radical scavenging activity in DPPH and ABTS assays (half-maximal inhibitory concentration [IC_50_] 16.0 ± 0.5 and 15.9 ± 0.4) than MRF did. In addition, the IRF 30% ethanol extract (200 μg/mL) significantly reduced the production of the inflammatory mediator NO by approximately 80% and inhibited iNOS, COX-2, phosphorylated (p)-IκB, and p-NF-κB activation compared with MRF. Moreover, IRF extract decreased the inflammatory cytokines tumor necrosis factor-α, interleukin (IL)-1β, and IL-6 compared with the MRF extract.

**Conclusions:**

This study revealed that IRF showed more beneficial effects than MRF did in LPS-stimulated RAW 264.7 macrophages, suggesting that IRF may be a useful anti-inflammatory agent.

**Electronic supplementary material:**

The online version of this article (10.1186/s12906-019-2496-6) contains supplementary material, which is available to authorized users.

## Background

*Rubus coreanus* fruit (RF), also called the Korean black raspberry, belongs to the family *Rosaceae* and is cultivated in the southern part of Korea, China, and Japan [1]. Immature RF (IRF) has been used to treat diabetes mellitus [2], while mature RF (MRF) has been used in anticancer, anti-inflammatory, and anti-fatigue treatments [3–5]. These potential health benefits of RF are attributed to different components including cyanidin 3-*O*-glucoside, cyanidin 3-*O*-xylosylrutinoside, cyanidin 3-*O*-rutinoside, ellagic acid (EA), and phenolics, such as gallic, protocatechuic, *p*-hydroxybenzoic, vanillic, syringic, salicylic, and caffeic acids. [6–8]. The levels of linoleic acid, citric acid, succinic acid, and sucrose decreased, while those of glucose and fructose increased during the maturation process [9].

Inflammatory response plays important roles in normal and pathological healing. Following an injury, various factors activate the immune system, leading to a local inflammatory response [10]. Pro-inflammatory cytokines, such as interleukin (IL)-6, IL-1β, and tumor necrosis factor (TNF), are stimulated and oxygen-centered free radical production is induced. The nuclear factor (NF)-κB transcription factor induces the transcription of pro-inflammatory genes and contributes to inflammation. Activated NF-κB is a stable trimeric complex of two NF-κB subunits (p65 and p50) and inhibitor of NF-κBα (IκBα). NF-κB induces key products such as pro-inflammatory cytokines, cyclooxygenase-2 (Cox-2), and inducible nitric oxide (NO) synthase (iNOS) [11]. Phosphorylation and degradation of IκB allows NF-κB to translocate to the nucleus [12, 13]. NOS isoenzymes [endothelial NOS (eNOS), neuronal NOS (nNOS), and iNOS] synthesize NO, and the production of high NO levels is characteristic of many inflammatory conditions [14]. Therefore, it is necessary to investigate the NOSs and NF-κB-induced transcription factors that induce pro-inflammatory cytokines, which subsequently cause inflammation-related diseases [15].

The anti-inflammatory effects of MRF have been previously published [16]; however, there are no reports on the effects of different harvest times and extraction solvents on the anti-inflammatory activity of RF. In this study, we investigated the effects of harvest time and various extraction solvents on the anti-inflammatory effects of RF as well as the potential involvement of NF-κB signaling in the mechanism of action of RF.

## Results

### Extraction yields of IRF and MRF according to extraction solvent

The yields of IRF extracts with 0, 30, and 70% ethanol were 15.22 ± 0.48, 17.33 ± 0.25, and 14.23 ± 0.19%, respectively, and the corresponding values of MRF extracts were 9.26 ± 0.66, 8.66 ± 0.25, and 8.07 ± 0.00%, respectively. IRF extract yield was higher than that of MRF, and the yield of IRF 30% ethanol extract was the highest.

### Antioxidant assay of IRF and MRF

The results of antioxidant assays are presented in Table 1. The results of DPPH and ABTS radical scavenging assays were expressed as IC_50_ values. IRF showed the highest DPPH radical scavenging activity (IC_50_ of 30% ethanol extract: 16.0 ± 0.5 μg/mL), while MRF showed the lowest (IC_50_ of 0% ethanol extract: 208.2 ± 17.1 μg/mL). Similar to DPPH results, IRF showed the highest ABTS radical scavenging activity (IC_50_ of 30% ethanol extract: 15.9 ± 0.4 μg/mL) and MRF showed the lowest (IC_50_ of 70% ethanol extract: 97.5 ± 2.7 μg/mL). The IRF extract showed significantly low IC_50_ values in DPPH and ABTS radical scavenging assays (*p* < 0.05), indicating that IRF exhibited the best antioxidant activity.

**Table 1:** IC_50_ value of antioxidant activity from 0, 30 and 70% ethanol extracts of mature and immature fruit of *Rubus coreanus* (*n* = 3)

Sample		DPPH (μg/mL)	ABTS (μg/mL)
MRF	0%	208.2 ± 17.1^a^	96.6 ± 8.3^a^
	30%	78.7 ± 2.9^c^	67.0 ± 1.6^b^
	70%	178.8 ± 2.0^b^	97.5 ± 2.7^a^
IRF	0%	24.0 ± 1.0^e^	21.6 ± 1.1^c^
	30%	16.0 ± 0.5^e^	15.9 ± 0.4^d^
	70%	32.2 ± 1.1^d^	27.6 ± 1.6^c^
Ascorbic acid		10.2 ± 0.3^e^	34.1 ± 0.3^c^

All values are means ± SD. Means with different letters are significantly different at *p* < 0.05 by Tukey’s multiple comparison tests. *MRF* mature *Rubus coreanus* fruit, *IRF* immature *Rubus coreanus* fruit

### IRF and MRF extracts inhibited NO production in LPS-stimulated RAW264.7 cells

**Fig. 1:**
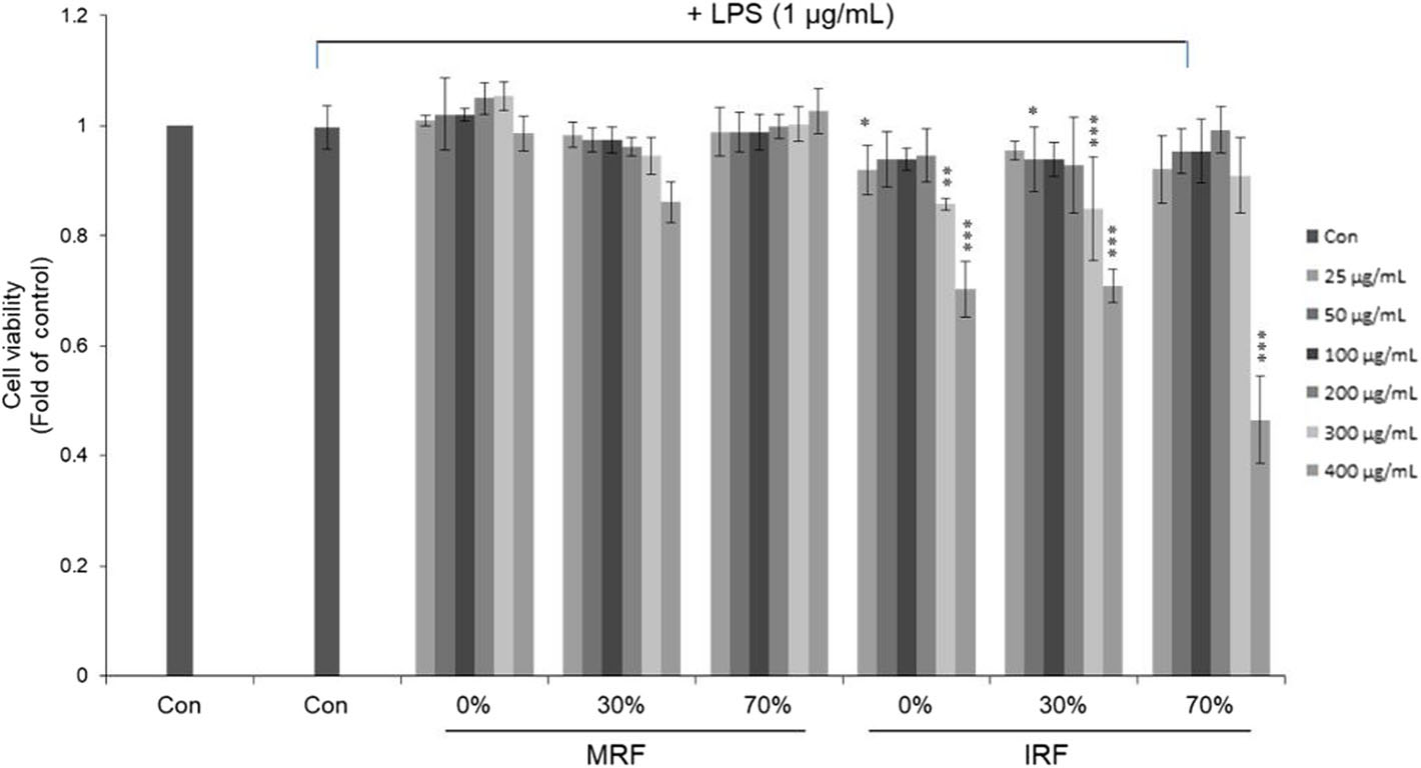
Cell viability of *Rubus coreanus* fruit (RF) extracts on lipopolysaccharide (LPS)-induced RAW264.7 cells. Cells were pretreated various concentrations of mature RF (MRF) and immature RF (IRF; 25, 50, 100, 200, 300, and 400 μg/mL) for 1 h and then 1 μg/mL LPS with extracts for 18 h. Cell viability was measured using MTS assay. All values are means ± SD, *n* = 3. ^*^p < 0.05, ^**^p < 0.01, and ^***^p < 0.001, comparing LPS control and each column

**Fig. 2:**
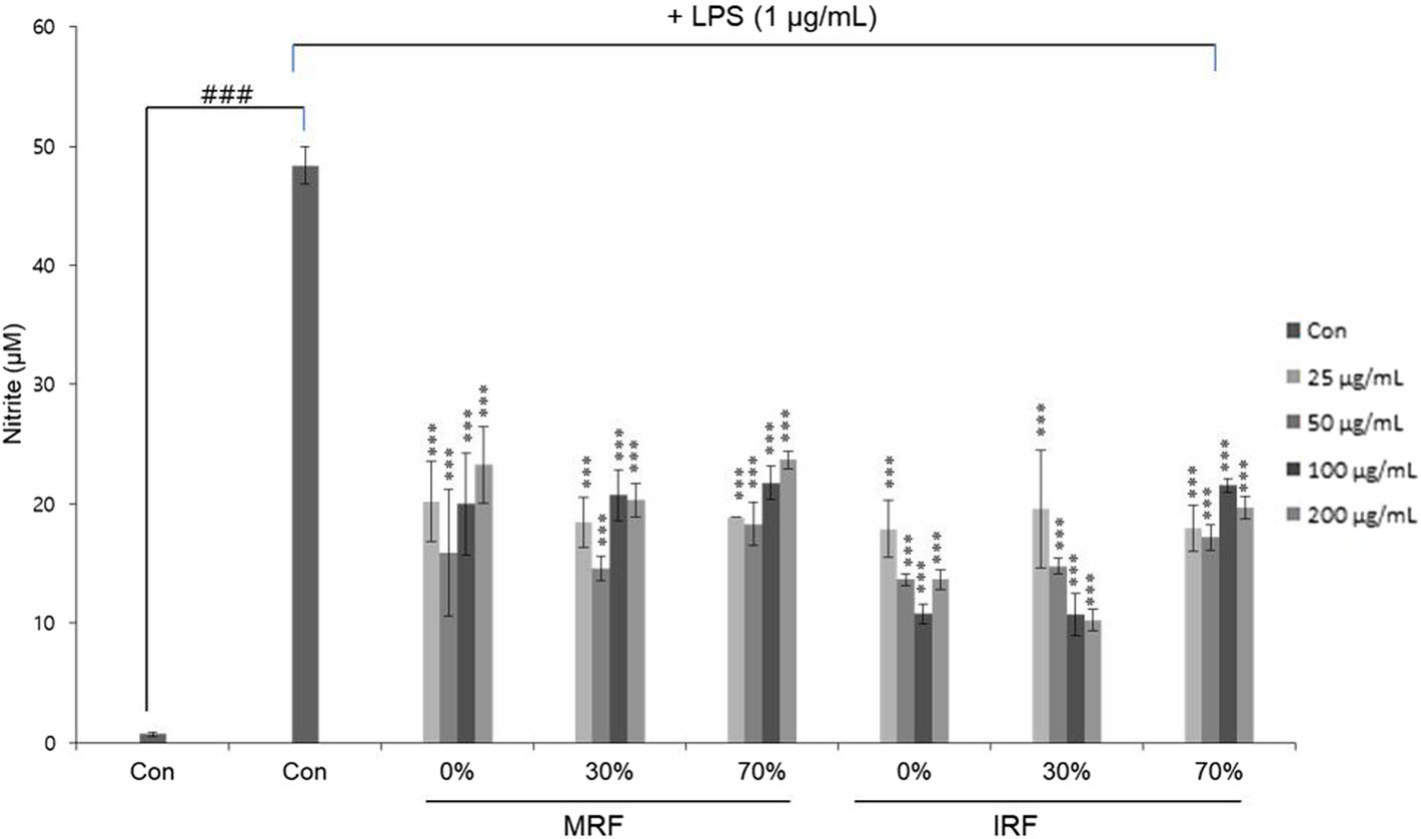
Effects of *Rubus coreanus* fruit (RF) extracts on nitric oxide (NO) production of lipopolysaccharide (LPS)-induced RAW264.7 cells. Cells were pretreated with various concentrations of mature RF (MRF) and immature RF (IRF; 25, 50, 100, and 200 μg/mL) for 1 h, followed by 1 μg/mL LPS for 18 h. NO production was measured using Griess reagent. All values are means ± SD, *n* = 3. . ^###^*p* < 0.001, comparing control and LPS control. ^***^p < 0.001, comparing LPS control and each concentration

Cell viability was determined after treatment with various concentrations of IRF and MRF extracts (0 to 400 μg/mL). The cell viability values were > 90% after exposure to up to 200 μg/mL of the extracts compared with the control (LPS treatment only, Fig. 1); however, the extracts affected cell viability at 400 μg/mL. All extract concentrations < 200 μg/mL were nontoxic to the cells and were therefore used in the subsequent assays. As shown in Fig. 2, NO production in LPS-stimulated RAW264.7 cells significantly decreased. We observed that different concentrations of IRF and MRF inhibited NO production in LPS-stimulated RAW264.7 cells. Specifically, IRF and MRF 30% ethanol extracts (200 and 50 μg/mL, respectively) reduced cell viability. Thus, the extracts were used at a concentration of 200 μg/mL in the next assay.

### IRF inhibits IL-6, IL-1β, and TNF-α levels in LPS-stimulated RAW264.7 cells

**Fig. 3:**
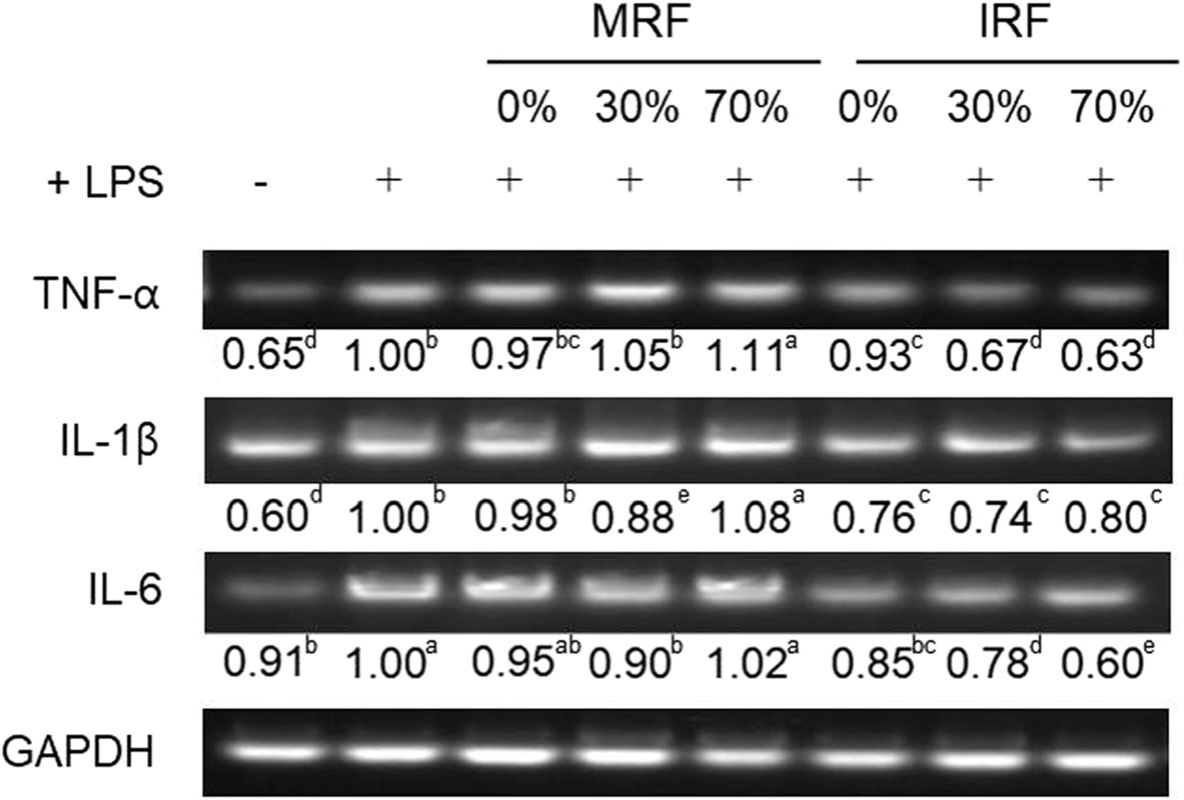
Anti-inflammatory effect of *Rubus coreanus* fruit (RF) extracts on mRNA expression. Representative bands of tumor necrosis factor (TNF)-α, interleukin (IL)-1β, and IL-6 are shown. Cells were treated with mature RF (MRF) and immature RF (IRF) at 200 μg/mL for 1 h, followed by 1 μg/mL LPS with extracts for 18 h. mRNA expressions were detected using reverse transcription-polymerase chain reaction (RT-PCR). The data depicted in the bend below as the means are an average of three similar and independent experiments. Means with different letters are significantly different at *p* < 0.05 by Tukey’s multiple comparison tests

We investigated the effect of IRF and MRF extracts on the expression levels of pro-inflammatory cytokines such as IL-6, IL-1β, and TNF-α. As shown in Fig. 3, RAW264.7 cells treated with LPS alone showed increased expression levels of IL-6, IL-1β, and TNF-α compared with the untreated cells. The expression levels of IL-6, IL-1β, and TNF-α were reduced in LPS-stimulated RAW264.7 cells following treatment with MRF extracts. IRF treatment also attenuated LPS-mediated overproduction of IL-6, IL-1β, and TNF-α. These data indicated that the inhibition of IL-6, IL-1β, and TNF-α by IRF may have been mediated by decreased NO production. The 30% ethanol extract of IRF showed a higher reduction in IL-6 and TNF-α levels.

### IRF extract inhibits expression of inflammatory-related proteins in LPS-stimulated RAW264.7 cells

**Fig. 4:**
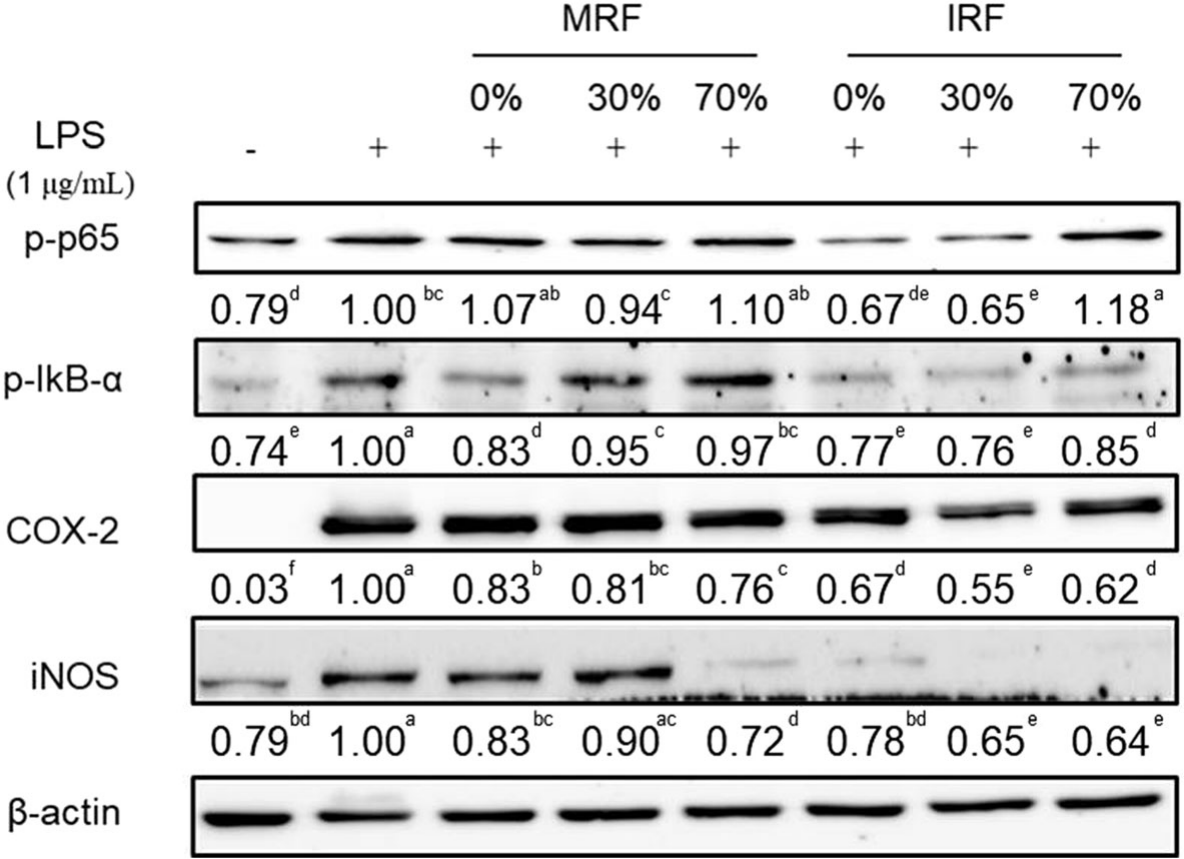
Anti-inflammatory effect of extracts from *Rubus coreanus* fruit (RF) on RAW264.7 protein expression. Cells were pretreated with 200 μg/mL mature RF (MRF) and immature RF (IRF) for 1 h and then induced with 1 μg/mL LPS for 18 h. Cells were lysed and proteins were employed by SDS-PAGE followed by Western blotting using primary antibodies targeting anti-p-p65, p-ikB-a, COX-2, iNOS and β-actin. . The data depicted in the bend below as the means are an average of three similar and independent experiments and gel images are of representative blots. Means with different letters are significantly different at *p* < 0.05 by Tukey’s multiple comparison tests

The NF-κB signaling pathway has been reported to be associated with the induction of gene expression of inflammatory mediators such as *NO*, *iNOS*, *COX-2*, *IL-6,* and *TNF-α* [11]. NF-kB activation is induced by the degradation of IkBα by IkB kinase-mediated phosphorylation and subsequent p65 nuclear translocation [17]. To determine whether the effects of 0, 30, and 70% ethanol extracts of IRF and MRF were mediated by the inflammatory-related proteins p-NF-κB (p65), p-IκB, COX-2, and iNOS in LPS-stimulated RAW264.7 cells, their protein expression was measured by western blotting (Fig. 4 and Additional file 1: Figure S1). LPS significantly enhanced p-p65, p-IκB, COX-2, and iNOS expression in RAW264.7 cells. In addition, RAW264.7 cells treated with LPS and IRF showed a higher reduction in expression of p-p65, p-IκB, COX-2, and iNOS than MRF-treated cells did. Furthermore, we showed that treatment of IRF 0 and 30% ethanol extract with LPS strongly suppressed inflammatory-related proteins. Our results suggest that IRF could negatively regulate inflammatory-related proteins in LPS-stimulated RAW264.7 cells and may therefore have anti-inflammatory effects on LPS-treated cells.

### Analysis of EA in IRF and MRF using HPLC

**Fig. 5:**
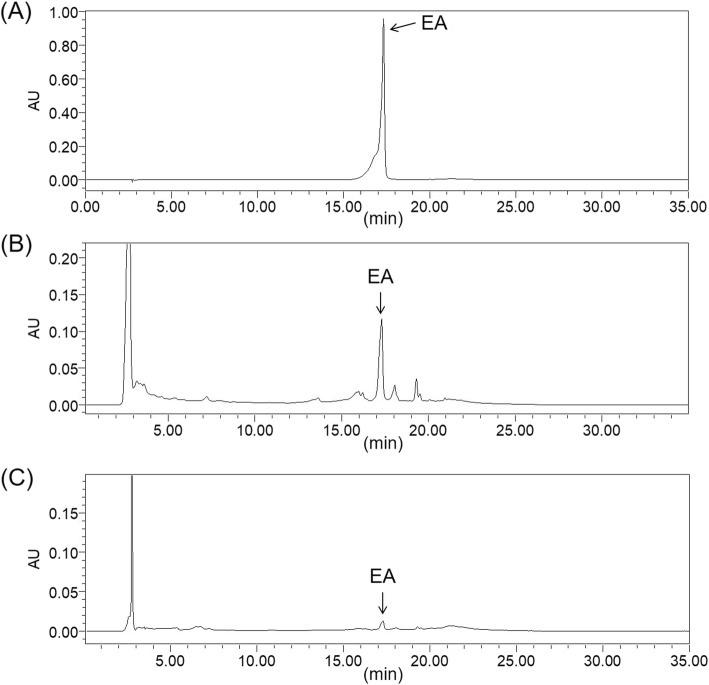
Liquid chromatography (LC) chromatograms of mature and immature *Rubus coreanus* fruit (MRF and IRF, respectively). Chromatogram: (**a**) Ellagic acid (EA, 200 μg/mL); (**b**) water extracts of MRF, 5 mg/mL; and (**c**) ethanol extracts of IRF, 5 mg/mL

EA is the main phenolic compound in RF [18, 19] and has beneficial effects, such as anti-inflammation, in mice and in damaged skin [20, 21]. Thus, we performed HPLC analysis to determine whether the inflammation inhibitory effect of IRF is attributed to EA content. The chromatograms of standard solution, MRF extract, and IRF extract are shown in Fig. 5. The EA contents of IRF and MRF were 0.75 and 0.10 mg/g dried weight, respectively.

## Discussion

RF is well known to have various pharmacological effects, including anticancer and anti-inflammatory [2–5, 16] activities, which are attributed to the phenolic compound anthocyanin and EA content [6, 9, 18]. However, the effect of different harvest times and extraction solvents on the anti-inflammatory effects of RF in LPS-stimulated RAW264.7 cells has not yet been examined. This is the first study to compare the anti-inflammatory effects of different solvent extracts of MRF and IRF in LPS-stimulated RAW264.7 cells. Total phenol and flavonoid contents were higher in 75% ethanol extract of MRF than in 25, 50, and 100% ethanol extract of MRF [22]. The anti-oxidant effect of 75% ethanol extracts of MRF and IRF was better than that of 25, 50, and 100% ethanol extracts in liver cells and DPPH radical scavenging activity [22, 23]. In addition, 30% ethanol extract of IRF has beneficial effect osteoporotic by pro-inflammation [24]. Based on these findings, water, 30% ethanol, and 70% ethanol were selected as extraction solvents in this study.

Injuries due to inflammation are mediated by oxygen-derived free radicals and high-energy oxidation, which cause toxic oxidation reactions in the cells [14]. In this study, the IC_50_ values in both ABTS and DPPH free radical scavenging assays were lower for IRF than for MRF. The 30% ethanol extract of IRF showed better antioxidant activity than MRF. RF contains anthocyanins and phenolic compounds in abundance [6], and their composition changes during maturation process. In particular, the contents of organic acids, amino acids, and phenolic compounds are reduced [25], while anthocyanin components formed that are glycosides attach to the phenolics [9]. Phenolic compounds are found in many fruits and vegetables, and they contribute to protective effects against oxidation in organisms [26]. EA is known to be a standard phenolic compound in RF and therefore, EA was selected as a standard component for the analysis. EA exerts potential activities such as anti-inflammatory and anticoagulatory effects in cardiac tissues of diabetic mice and UV–B irradiation-induced inflammation model [20, 21]. In the present study, IRF contained a higher quantity of EA than MRF did, indicating that the EA component in IRF inhibited the production of pro-inflammatory cytokines and inflammation-related factors in LPS-stimulated RAW264.7 cells. Raw 264.7 macrophages perform major functions such as initiation, maintenance, and resolution of inflammatory precess [27]. Our findings showed that IRF efficaciously inhibited p65 NF-κB inflammatory response via cytokine genes in LPS-stimulated RAW264.7 cells. Treatment of RAW264.7 cells with LPS stimulates toll-like receptor 4 and induces the release of pro-inflammatory cytokines necessary to activate immune response [28]. We demonstrated the inhibition of pro-inflammatory factors TNF-α, IL-1β, and IL-6 by IRF. In a previous study, 100% ethanol extract of IRF reduced pro-inflammatory cytokines more effectively than MRF extracts did [16]. However, 0 and 30% ethanol extracts of IRF reduced pro-inflammatory cytokines to a lesser extent than 100% ethanol extract did. Pro-inflammatory cytokine actions involve the activation of NF-κB. Cytokines may be responsible for the induction of COX-2 and iNOS. Furthermore, iNOS produces NO to cause inflammation [14]. In the present study, treatment of LPS-stimulated RAW264.7 cells with 0 and 30% ethanol extracts of IRF reduced NO and inflammatory-related proteins, and these effects were less potent than those induced by the 100% ethanol extract (similar to effects on pro-inflammatory cytokines) [16]. The extract with the highest polyphenol content exhibited the highest antioxidant capacity [6]. A previous study reported that the ethyl acetate fraction of RF, which had the highest polyphenol content, showed the highest NO reduction rate [8]. These results indicate that the ethanol extract of IRF, which had antioxidant ability, showed better anti-inflammatory effects than MRF.

Our results confirmed that the anti-inflammatory effects of 0 and 30% ethanol extracts of IRF, which were mediated by reduction of NF-κB pathway activity via cytokine inhibition in LPS-stimulated RAW264.7 cells, were higher than that of the 70% ethanol extracts of IRF and MRF. This effect may have been mediated by the anti-inflammatory effects of EA in IRF. Therefore, 0 and 30% ethanol extracts of IRF have the potential to be used as therapeutic agents for inflammation-related diseases.

## Conclusion

Our results revealed that IRF inhibited inflammatory responses induced by NF-kB signaling and pro-inflammatory cytokines (TNF-α, IL-1β, and IL-6). In addition, the EA content of RF, which is known to have anti-inflammatory activity, was higher in IRF than in MRF. This study was the first to describe the detailed inflammatory mechanisms of IRF and MRF extracted using different ethanol concentrations. Furthermore, these results suggest that IRF could be used as a dietary supplement with potential benefits in treating inflammation-related diseases.

## Materials and methods

### Preparation of materials, extracts, and yields

*R. coreanus* grown for 30 and 45–70 days post-bloom (IRF and MRF, respectively) were collected from Gwang Yang in South Korea in 2017. The code and production income sales report number of the plant material (09-01-0026 and 09–004–2005-1) was deposited at the National Forest Seed and Variety Center and identified by PhD. Seok Cheol Suh (Gwangyang Agricultural Technology Center) [29]. In addition, the voucher specimen (NIBRVP0000180126) was deposited at National institute of biological resources [30]. Crushed RF samples were stored at − 20 °C before extraction and were hot-air dried, crushed, and subsequently extracted. IRF and MRF were reflux extracted three times with different concentrations of ethanol (0, 30, and 70%). The sample extracts were filtered, vacuum evaporated, freeze-dried, stored at − 70 °C, and then used in each experiment. The weight of dried samples (W_sample_) and freeze-dried extracts (W_extracts_) were measured and the yield was calculated using the following equation:

$$ \mathrm{Yield}\ \left(\%\right)={\mathrm{W}}_{\mathrm{extracts}}\ \left(\mathrm{g}\right)/{\mathrm{W}}_{\mathrm{sample}}{\left(\mathrm{g}\right)}^{\ast }\ 100 $$

### 2,2-Diphenyl-1-picrylhydrazyl scavenging assay

2,2-Diphenyl-1-picrylhydrazyl (DPPH) scavenging assay was carried out using the Müller method [31] with modifications. Sample stock solutions (10 mg/mL) were diluted to final concentrations of 10 to 500 μg/mL in ethanol and mixed with 0.25 mM DPPH solution. After 30 min, the absorbance at 515 nm, which is the maximum absorbance of DPPH, was recorded as Abs_sample_ using an ultraviolet/visible (UV/VIS) spectrophotometer (BioTek Instruments, Inc., Winooski, USA). A blank experiment was also carried out using the same procedure with ethanol plus the extract solution, and the absorbance was recorded as Abs_blank_. The radical scavenging activity (RSA) of each solution was then calculated as a percentage according to the following equation:

$$ \mathrm{RSA}\ \left(\%\right)=\left[\left({\mathrm{Abs}}_{\mathrm{sample}}-{\mathrm{Abs}}_{\mathrm{blank}}\right)/{\mathrm{Abs}}_{\mathrm{blank}}\right]\times 100 $$

The positive control was ascorbic acid, and each value was expressed as the half-maximal inhibitory concentration (IC_50_), which is the concentration that scavenges 50% of the free radicals.

### 2,2′-Azino-bis(3-ethylbenzothiazoline-6-sulphonic acid scavenging assay

The 2,2′-Azino-bis(3-ethylbenzothiazoline-6-sulphonic acid (ABTS) scavenging activity of the extracts was examined against ABTS radical cation generated using chemical method [32]. Sample stock solutions (10 mg/mL) were diluted to final concentrations of 50 to 300 μg/mL in water. ABTS was dissolved in water to a concentration of 7 mM. The radical cations were produced by reacting the stock solution with 2.45 mM of potassium peroxodisulfate and allowing the mixture to stand at − 4 °C for 4 h before using the ABTS^+^ solution. The absorbance at 734 nm of the mixture of ABTS^+^ and various concentrations of the extracts, recorded as Abs_sample_, was measured using a UV/VIS spectrophotometer. A blank experiment was also carried out by the same procedure using water plus the extract solution, and the absorbance was recorded as Abs_blank_. The RSA of each solution was calculated as a percentage according to the following equation:

$$ \mathrm{RSA}\ \left(\%\right)=\left[\left({\mathrm{Abs}}_{\mathrm{blank}}-{\mathrm{Abs}}_{\mathrm{sample}}\right)/{\mathrm{Abs}}_{\mathrm{blank}}\right]\times 100. $$

The positive control was ascorbic acid and each value was expressed as the IC_50_.

### Cell culture

RAW264.7 macrophages were cultured in Dulbecco’s modified Eagle’s medium (DMEM) supplemented with 5% fetal bovine serum (FBS), 1% penicillin-streptomycin (Gibco BRL, Burlington, ON, Canada) at 37 °C in a 5% CO_2_ incubator. The medium was replaced every 2 days for subculture.

### Measurement of cell viability

To evaluate the effects of the extracts on cell viability, a CellTiter 96® AQ_ueous_ One Solution cell proliferation assay (3-(4,5-dimethylthiazol-2-yl)-5-(3-carboxymethoxyphenyl)-2-(4-sulfophenyl)-2H-tetrazolium [MTS]) was performed. The cells were plated in 96-well plates at a density of 1.0 × 10^5^ cells/well. After 24 h, the medium was replaced with serum-free medium, and the cells were treated with various concentrations (0 to 400 μg/mL) of IRF and MRF extracts for 1 h, followed by 1 μg/mL of lipopolysaccharide (LPS). After 18 h, 100 μL of DMEM containing MTS was added to each well and incubated at 37 °C for 1 h. The medium was removed and 120 μL of dimethyl sulfoxide was added, and the plate was shaken for 5 min at room temperature to dissolve the precipitated formazan. The absorbance of the resultant solution was measured at 490 nm using a multi-plate reader (BioTek Instruments, Inc., Winooski, USA).

### Measurement of NO production

To determine the effect of IRF and MRF extracts on NO production, Griess reagent (Promega) was used according to the manufacturer’s instructions. The cells were plated in 96-well plates at a density of 1.0 × 10^5^ cells/well for 24 h. The medium was replaced with serum-free medium, and the cells were treated with various concentrations (0 to 400 μg/mL) of MRF and IRF extracts for 1 h, followed by 1 μg/mL of LPS. After 18 h, equal amounts of the culture medium and Griess reagent were reacted in a 96-well plate for 10 min, and the absorbance was measured at 540 nm using a multi-plate reader.

### mRNA analysis using reverse transcription polymerase chain reaction

RNA from RAW264.7 macrophages was isolated using 1 mL of TRIzol (Ambion, MA, USA). cDNA was synthesized from 1 μg of total RNA using Reverse Transcriptase Premix (Intron, Seongnam-Si, Korea). Polymerase chain reaction (PCR) analysis was performed with 20 ng of cDNA using the Maxime PCR PreMix kit (*i*-Taq, Intron) to detect TNF-α, IL-6, IL-1β, and glyceraldehyde 3-phosphate dehydrogenase (GAPDH). The forward and reverse primers are presented in Table 2. Reactions were performed on a PCR System, and the thermal profile settings were 50 °C for 2 min and 95 °C for 2 min. In addition, 30 cycles were performed for TNF-α, IL-6, IL-1β, and GAPDH (at 94 °C for 300 s, 58 °C for 30 s, and 72 °C for 30 s). After PCR, electrophoresis was performed using a 1% agarose gel, visualized using loading STAR (Dyne bio) staining, and detected using a UV spectrometer (Davinch-K, Korea).

**Table 2:** mRNA of forward and reverse primers sequence

Primer name		Sequence	Size (bp)
TNF-α	Forward	5′-CACACTCAGATCATCTTCTCAA-3′	198
	Reverse	5′-TTGAAGAGAACCTGGGAGTAG-3′	
IL-1β	Forward	5′-GTATCACTCATTGTGGCTGTG-3′	329
	Reverse	5′-ATTTTGTCGTTGCTTGGTTCTC-3′	
IL-6	Forward	5′-ATTACACATGTTCTCTGGGAAG-3’	312
	Reverse	5′-TTTTACCTCTTGGTTGAAGATATG-3’	
GAPDH	Forward	5′-AAAAGGGTCATCATCTCCGC-3’	432
	Reverse	5′-CTTCTTGATGTCATCATACTTGG-3’	

### Western blotting

RAW264.7 macrophages were collected and lysed in ice-cold radioimmunoprecipitation assay buffer (Cell signaling, MA, USA) for 30 min. The protein contents were measured by Bradford protein assay (Bio-Rad, CA, USA). Total proteins (20 μg) were subjected to sodium dodecyl sulfate-polyacrylamide gel electrophoresis and electro-transferred onto polyvinylidene difluoride membrane (Millipore, Darmstadt, Germany). The membranes were blocked with 5% nonfat-milk for 30 min at room temperature and incubated in a 1:1000 dilution of the primary antibody overnight at 4 °C. The primary antibodies used were phospho Nf-κB p-65 (p-p65), phospho-IκB-α (p- IκB-α), Cox-2, iNOS, and β-actin. All antibodies were purchased from Cell Signaling (USA). The membranes were washed three times with Tris-buffered saline plus Tween 20 for 10 min each time. After that, the membranes were incubated in a 1:2000 dilution of horseradish peroxidase-conjugated secondary antibody for 1 h at room temperature. The protein reactions were detected using enhanced chemiluminescence reagent (Bio-Rad, CA, USA) and chemi-luminator (Davinch-K, Seoul, Korea).

### High-performance liquid chromatography analysis of EA

The analysis of EA was performed using a high-performance liquid chromatography (HPLC) system from Waters 2795 series (TX, USA), equipped with a diode array detector from Waters 2495 series (TX, USA). HPLC separation of EA for quantitative analysis was performed using a reverse phase system. The components of the chromatographic profile of IRF and MRF extracts were identified by comparison with retention times (RT) of peaks in the EA standard solution. An INNO C18 (4.6 × 250 mm, 5 μm) column was used with a mobile phase comprising 0.1% phosphoric acid in water (A) and methanol (B) for EA. The elution program was a modification of the method by Chae et al. [18] with a gradient solvent system (B from 30 to 70% for 25 min). UV detection was performed at 370 nm. The injection volume was 10 μL, the flow rate was 1.0 mL/min, and temperature was 35 °C.

### Statistical analysis

All experimental results are presented as the means ± standard deviation (SD) of three independent experiments. The statistical significance of differences in this study was calculated using a one-way analysis of variance using Tukey’s test (GraphPad Prism 5.02).

## Additional file

Additional file 1: **Figure S1.** Anti-inflammatory effect of extracts from *Rubus coreanus* fruit (RF) on RAW264.7 protein expression. Cells were pretreated with 200 μg/mL mature RF (MRF) and immature RF (IRF) for 1 h and then induced with 1 μg/mL LPS for 18 h. Cells were lysed and proteins were employed by SDS-PAGE followed by Western blotting using primary antibodies targeting anti-p-p65, p-ikB-a, COX-2, iNOS and β-actin. (DOCX 414 kb)

## Abbreviations

COX: Cyclooxygenase
EA: Ellagic acid
IL: Interleukin
IRF: Immature *Rubus coreanus* fruit
LPS: Lipopolysaccharide
MRF: *Rubus coreanus* fruit
NF-kB: Nuclear factor-kB
NO: Nitric oxide
NOS: Nitric oxide synthase
TLR: Toll-like receptor
TNF: Tumor necrosis factor

## References

[1] Ku CS, Mun SP (2008). Antioxidant activities of ethanol extracts from seeds in fresh Bokbunja (Rubus coreanus Miq.) and wine processing waste. Bioresour Technol.

[2] Seo YC, Choi WY, Kim JS, Yoon CS, Lim HW, Cho JS, hee Ahn J, Lee HY (2011). Effect of ultra high pressure processing on immuno-modulatory activities of the fruits of Rubus coreanus Miquel. Innovative Food Sci Emerg Technol.

[3] Choung MG, Lim JD (2012). Antioxidant, anticancer and immune activation of anthocyanin fraction from Rubus coreanus Miquel fruits (Bokbunja). Korean J Med Crop Sci.

[4] Shin JS, Cho EJ, Choi HE, Seo JH, An HJ, Park HJ, Cho YW, Lee KT (2014). Anti-inflammatory effect of a standardized triterpenoid-rich fraction isolated from Rubus coreanus on dextran sodium sulfate-induced acute colitis in mice and LPS-induced macrophages. J Ethnopharmacol.

[5] Jung KA, Han DS, Kwon EK, Lee CH, Kim YE (2007). Antifatigue effect of Rubus coreanus Miquel extract in mice. J Med Food.

[6] Ju HK, Cho EJ, Jang MH, Lee YY, Hong SS, Park JH, Kwon SW (2009). Characterization of increased phenolic compounds from fermented Bokbunja (Rubus coreanus Miq.) and related antioxidant activity. J Pharm Biomed Anal.

[7] Im SE, Nam TG, Lee HJ, Han MW, Heo HJ, Koo SI, Lee CY, Kim DO (2013). Anthocyanins in the ripe fruits of Rubus coreanus Miquel and their protective effect on neuronal PC-12 cells. Food Chem.

[8] Lim JW, Hwang HJ, Shin CS (2012). Polyphenol compounds and anti-inflammatory activities of Korean black raspberry (Rubus coreanus Miquel) wines produced from juice supplemented with pulp and seed. J Agric Food Chem.

[9] Kim HS, Park SJ, Hyun SH, Yang SO, Lee JH, Auh JH, Kim JH, Cho SM, Marriott PJ, Choi HK (2011). Biochemical monitoring of black raspberry (Rubus coreanus Miquel) fruits according to maturation stage by 1H NMR using multiple solvent systems. Food Res Int.

[10] Koh TJ, DiPietro LA. Inflammation and wound healing: the role of the macrophage. Expert Rev Mol Med. 2011;13:e23. 10.1017/S1462399411001943 PMC3596046 21740602

[11] Tak PP, Firestein GS (2001). NF-κB: a key role in inflammatory diseases. J Clin Invest.

[12] Jones B, Heldwein K, Means T, Saukkonen J, Fenton M (2001). Differential roles of toll-like receptors in the elicitation of proinflammatory responses by macrophages. Ann Rheum Dis.

[13] Chow JC, Young DW, Golenbock DT, Christ WJ, Gusovsky F (1999). Toll-like receptor-4 mediates lipopolysaccharide-induced signal transduction. J Biol Chem.

[14] Cuzzocrea S, Riley DP, Caputi AP, Salvemini D (2001). Antioxidant therapy: a new pharmacological approach in shock, inflammation, and ischemia/reperfusion injury. Pharmacol Rev.

[15] Huang M-Y, Tu CE, Wang SC, Hung YL, Su CC, Fang SH, Chen CS, Liu PL, Cheng WC, Huang YW (2018). Corylin inhibits LPS-induced inflammatory response and attenuates the activation of NLRP3 inflammasome in microglia. BMC Complement Altern Med.

[16] Yang HM, Sm O, Lim SS, Shin HK, Oh YS, Kim JK (2008). Antiinflammatory activities of Rubus coreanus depend on the degree of fruit ripening. Phytother Res.

[17] Serasanambati M, Chilakapati SR (2016). Function of nuclear factor kappa B (NF-kB) in human diseases-a review. S Indian J Biol Sci.

[18] Chae K, Son R, Park S, Kim K, Lee T, Kwon J. Analytical method validation of ellagic acid as a marker compound for the standardization of black raspberry extract as a functional ingredient. Food Eng Prog. 2014;18(4):355–58.

[19] Yoon I, Cho JY, Kuk JH, Wee JH, Jang MY, Ahn TH, Park KH (2002). Identification and activity of antioxidative compounds from Rubus coreanum fruit. Korean J Food Sci Technol.

[20] Chao PC, Hsu CC, Yin MC (2009). Anti-inflammatory and anti-coagulatory activities of caffeic acid and ellagic acid in cardiac tissue of diabetic mice. Nutr Metab.

[21] Bae JY, Choi JS, Kang SW, Lee YJ, Park J, Kang YH (2010). Dietary compound ellagic acid alleviates skin wrinkle and inflammation induced by UV-B irradiation. Exp Dermatol.

[22] Kim KA, Kwon JW, Kim YS, Park PJ, Chae KS (2015). Antioxidant activities of ethanol extracts from different parts of the black raspberry (Rubus occidentalis) obtained using ultra-sonication. Korean J Food Sci Technol.

[23] Bhandary B, Lee H, Back H, Park S, Kim M, Kwon J, Song J, Lee H, Kim H, Chae S (2012). Immature rubus coreanus shows a free radical-scavenging effect and inhibits cholesterol synthesis and secretion in liver cells. Indian J Pharm Sci.

[24] Kim HJ, Sim DS, Sohn EH (2014). Anti-osteoporotic effects of unripe fructus of Rubus coreanus Miquel in osteoblastic and osteoclastic cells. Korean J Plant Res.

[25] Kim JM, Shin MS (2011). Characteristics of Rubus coreanus Miq. Fruits at different ripening stages. Korean J Food Sci Technol.

[26] Rice-Evans C, Miller N, Paganga G (1997). Antioxidant properties of phenolic compounds. Trends Plant Sci.

[27] Fujiwara N, Kobayashi K (2005). Macrophages in inflammation. Curr Drug Targets Inflamm Allergy.

[28] Lu YC, Yeh WC, Ohashi PS (2008). LPS/TLR4 signal transduction pathway. Cytokine.

[29] Kim S, Chung H, Han J (2007). A new high productivity Rubus coreanus Miq. Cultivar, “Jung-Keum 1”. Korean J Breed Sci.

[30] Jo JL, Kil HJ, Woo BH, Choi SH, Park CH, Kim CM (2010). The Documentation of Type and Voucher Specimens of Indigenous Species of Korea. Biological Resources Research Department National Institute of Biological Resources.

[31] Müller L, Fröhlich K, Böhm V (2011). Comparative antioxidant activities of carotenoids measured by ferric reducing antioxidant power (FRAP), ABTS bleaching assay (αTEAC), DPPH assay and peroxyl radical scavenging assay. Food Chem.

[32] Miliauskas G, Venskutonis P, Van Beek T (2004). Screening of radical scavenging activity of some medicinal and aromatic plant extracts. Food Chem.

