# Supplementary material for: Differences in anti-inflammatory effect of immature and mature of Rubus coreanus fruits on LPS-induced RAW 264.7 macrophages via NF-κB signal pathways
Source: BMC Complement Altern Med. 2019 Apr 25;19:89. doi: 10.1186/s12906-019-2496-6 (PMC6485102; doi:10.1186/s12906-019-2496-6)
Supplement: Supplementary file 1 — Figure S1. Anti-inflammatory effect of extracts from Rubus coreanus fruit (RF) on RAW264.7 protein expression. Cells were pretreated with 200 μg/mL mature RF (MRF) and immature RF (IRF) for 1 h and then induced with 1 μg/mL LPS for 18 h. Cells were lysed and proteins were employed by SDS-PAGE followed by Western blotting using primary antibodies targeting anti-p-p65, p-ikB-a, COX-2, iNOS and β-actin. (DOCX 414 kb) [file 12906_2019_2496_MOESM1_ESM.docx]

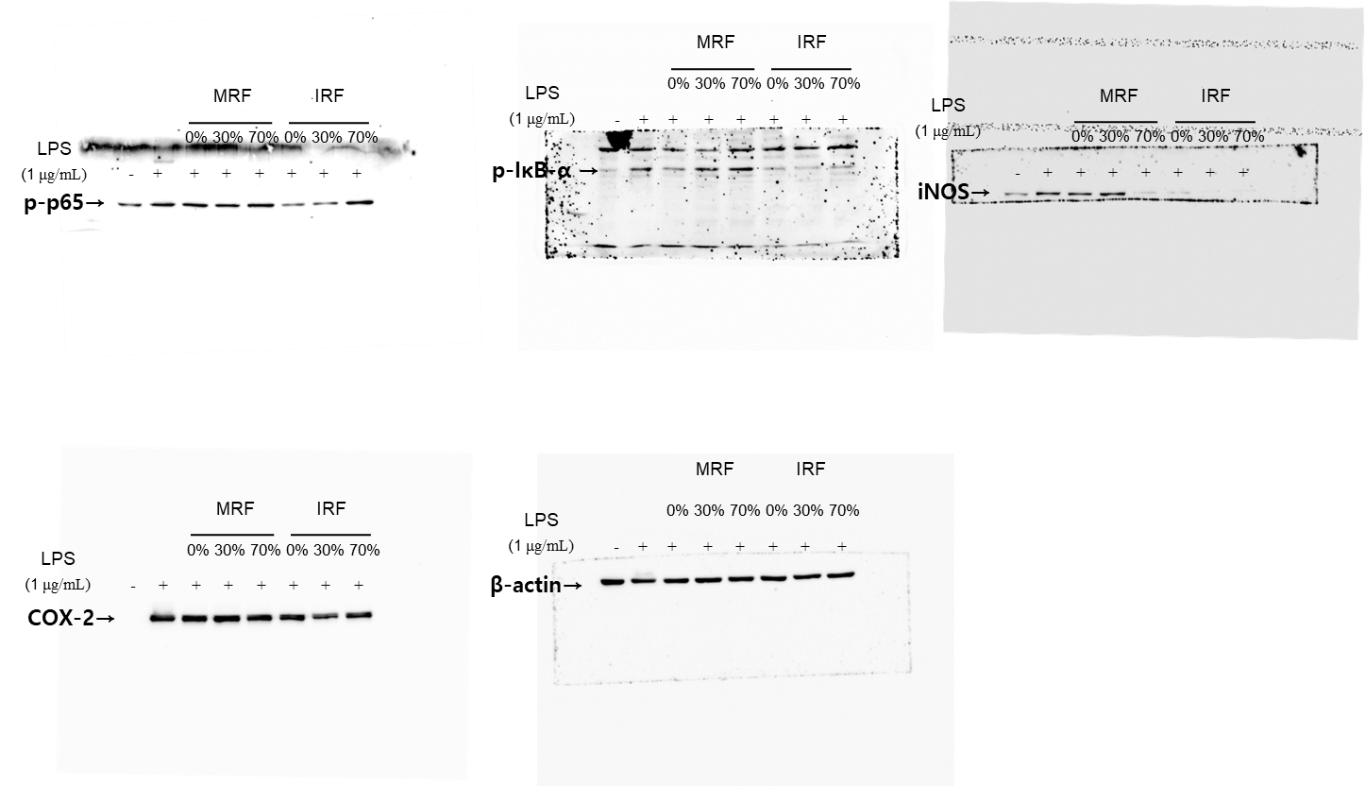


**Additional Figure 1. Anti-inflammatory effect of extracts from *Rubus coreanus* fruit (RF) on RAW264.7 protein expression.** Cells were pretreated with 200 μg/mL mature RF (MRF) and immature RF (IRF) for 1 h and then induced with 1 μg/mL LPS for 18 h.  Cells were lysed and proteins were employed by SDS-PAGE followed by Western blotting using primary antibodies targeting anti-p-p65, p-ikB-a, COX-2, iNOS and β-actin. .
